# Brain aerobic glycolysis is stable during adulthood: Direct evidence from cross-brain blood sampling in 239 healthy adults

**DOI:** 10.1177/0271678X251399122

**Published:** 2026-01-08

**Authors:** Jennifer S Duffy, Philip N Ainslie, Peter Rasmussen, David B MacLeod, Travis D Gibbons

**Affiliations:** 1 Centre for Heart, Lung and Vascular Health, School of Health and Exercise Science, University of British Columbia—Okanagan, Kelowna, BC, Canada; 2 Genmab A/S, Valby, Denmark; 3 Department of Anesthesiology, Duke University Medical Center, Durham, NC, USA; 4 Department of Biological Sciences, Northern Arizona University, Flagstaff, AZ, USA

**Keywords:** Aerobic glycolysis, aging brain, arteriovenous difference, cerebral metabolism, neurodegeneration

## Abstract

The brain is a highly metabolic organ primarily fueled by glucose, and it is well established that a decline in cerebral glucose metabolism accompanies neurodegenerative disease. Recent work using positron emission tomography (PET) has demonstrated that cerebral glucose metabolism is also reduced in healthy aging which commences as early as 20 years of age, driven almost exclusively by reductions in non-oxidative glucose metabolism, that is, aerobic glycolysis. Given the historical variability and assumptions in PET-based interpretations regarding cerebral glucose metabolism with aging, we aimed to establish whether a drop in global aerobic glycolysis is truly a component of healthy aging in adulthood using direct measures of carbohydrates and oxygen across the human brain via invasive cross-brain blood sampling. We accumulated resting data from 17 studies comprising 239 healthy adults aged 19–45 years and show that aerobic glycolysis remains stable during aging based on a regression analysis of the arteriovenous differences for oxygen, glucose, and lactate, and their resultant ratios (oxygen glucose/carbohydrate indices). The direct cross-brain data presented here indicate that declining brain aerobic glycolysis is not a feature of normal brain aging during early-mid adulthood.

## Introduction

The human brain is a gluttonous organ as it accounts for 2% of our body mass yet contributes to 20% of our resting energy demands, almost exclusively provided by glucose.^1,2^ Throughout childhood, the brain undergoes extensive growth in volume and mass accompanied by synapse development and reorganization.^3,4^ After childhood, the adult brain undergoes decreases in mass and volume,^5^ synaptic density,^6^ and a reduction in cerebral blood flow^7,8^ and glucose metabolism,^9,10^ with these aging outcomes exaggerated in neurodegenerative diseases.^9–12^ It is uncertain whether a drop in brain glucose metabolism is a causal factor in the development of cognitive decline and overt neurodegenerative disease.^10^ It has been demonstrated that glucose hypometabolism occurs well in advance of any measurable cognitive decline or symptoms,^13–16^ lending to the possibility that metabolic dysfunction early in adulthood may be the canary in the coal mine when it comes to neurodegenerative disease. Understanding when—and in what way—the decline in cerebral metabolism of glucose (and other substrates) occurs throughout the lifespan provides critical insight to distinguish normal from pathological brain aging.

Positron emission tomography (PET) imaging has shown that a drop in glucose metabolism with healthy aging is driven almost exclusively by declining aerobic glycolysis (AG),^17^ the metabolism of glucose without oxygen despite adequate oxygen availability.^18^ Interestingly, it was shown that the bulk of this decline occurs between the ages of 20 and 40 years old,^17^ making it further unclear whether glucose hypometabolism is an early warning sign of cognitive decline. This is a clinically meaningful finding as AG, while energetically inefficient, is a vital process during brain activation,^19^ learning,^20^ and memory formation,^21^ and is highest in the early stages of life when synaptic plasticity and growth are occurring at rapid rates.^22^ In the elderly there is evidence that a drop in AG accompanies neurodegeneration-related symptoms,^23^ which is in accordance with the notion that neurodegeneration occurs alongside declining brain glucose metabolism.

Since aging is the biggest risk factor for neurodegenerative disease,^24^ and a drop in glucose metabolism is a primary feature of such diseases, it is critical to reconcile whether a drop in AG (and to what degree) is an obligatory part of normal aging or underlies pathological development. As it stands, our current methodological techniques and study designs (primarily PET-imaging) have been unable to consistently identify brain metabolic changes, with conflicting results reported on the fate of cerebral glucose metabolism with age (reviewed in Cunnane et al.^10^). Herein, we present data amalgamated from laboratories across the world that have used a direct approach to quantify whole brain AG. This fundamental approach employs concurrent blood sampling from the internal jugular bulb and arterial circulation, allowing for the direct calculation of cross-brain oxygen, glucose, and lactate extraction. AG can then be quantified by determining the arteriovenous difference of glucose, lactate, and oxygen and calculating the ratio of oxygen-to-glucose or carbohydrate extraction (OGI/OCI), reflecting glucose/carbohydrate metabolism in proportion to oxygen. Earlier work using direct blood sampling demonstrated a non-significant increase in oxygen-glucose metabolic ratios between a young (20 years) and older (71 years) cohort,^9,25^ leaving a dearth of data in the age range which has recently been deemed the period for reductions in AG.^17^ In 239 healthy humans ranging from 19 to 45 years old, we report stability of AG, with preliminary evidence that this persists into the elderly (age 60+ years).

## Methods

### Study and participant details

We collected both arterial and internal jugular venous bulb blood sample data from 17 studies in healthy humans. Eight of these studies (two unpublished)^26–31^ were data collected from University of British Columbia (UBC), Canada, seven from the Muscle Research Laboratory (MRL), Copenhagen, Denmark^32–39^; and two from the Department of Anesthesiology, Duke University; USA (both unpublished studies). The reasons for including this combination of studies were due to the consistent methodology, techniques, researchers, and physicians. We initially compiled data from eight studies conducted in healthy humans by our lab group at UBC, performed preliminary analyses, and generated our hypothesis based on these subjects. We then reached out to researchers known to have conducted arteriovenous studies using the same techniques (see *Method Details*), resulting in a relatively homogenous dataset. Although the blood gas analyzer (ABL) model differed between UBC and MRL, we believe this would have a minimal effect on data variability. Notably, the two Duke University studies were conducted by the same physician responsible for the standardized instrumentation in the UBC studies. Across all studies, only three physicians placed the arterial and jugular lines, helping to further minimize variability in research techniques.

All studies recruited only healthy adults with no known cardiovascular or neurological diseases between the ages of 19 and 45 years old (mean 26.4 ± 5.1 years). In 5 of 17 studies, accounting for 49 participants (20%), maximal aerobic power data was available and averaged 48 ml/min/kg indicating good-to-excellent fitness.^40^ One study investigated elderly subjects aged 66.4 ± 6.7 years old (*n* = 9).^34^ All studies were approved by the local ethics committees (University of British Columbia Clinical Research Ethics Board, the Ethics Committee of Copenhagen, and the Institutional Review Board of Duke University Medical Center), carried out in accordance with the Declaration of Helsinki, and informed consent was obtained from all participants. More details on each study are included in the Supplemental Material (Table S1).

### Method details

#### Cross-brain blood sampling

In each study, arterial and central venous catheters were placed with the participant supine, using a sterile technique under local anesthesia, and assisted via the use of ultrasound guidance. To accurately isolate cerebral metabolism it is critical that blood sampled from the internal jugular vein comprises no contamination from extra-cerebral blood. This was done via cranial advancement (~15 cm) of the catheter, placing the catheter tip in the jugular bulb.^41^ Following this approach, it has been demonstrated to lead to >97% sampling of venous blood isolated from the brain.^42^ Blood samples from the artery (typically brachial or radial) and internal jugular vein were drawn simultaneously and immediately analyzed using a commercial blood gas analyzer (ABL FLEX Radiometer), which measures oxygen, glucose, and lactate concentrations.

#### Establishing trends of substrate arteriovenous (A-V) differences (extraction) with age

Resting baseline samples from each study were used to calculate metrics of aerobic glycolysis with healthy aging. First, the arteriovenous (A-V) difference was calculated for oxygen, glucose, and lactate in mmol/L. Extraction fractions were then determined as per equation (1) for oxygen, glucose, and lactate. A linear regression of extraction fractions (%) with age was established and plotted to visualize the change of substrate metabolism with aging.

Oxygen A-V differences were then divided by 6 to be expressed as glucose equivalents. Similarly, lactate A-V differences we divided by 2 to account for three carbons provided by lactate relative to six provided by glucose, and summed with the A-V glucose differences to generate the A-V carbohydrate difference. A linear regression of glucose-scaled oxygen, glucose, and total carbohydrate A-V differences with age was established and plotted to visualize the change of substrate metabolism relative to oxygen consumption.

#### Quantification of aerobic glycolysis

In this work we quantify AG using the ratio of oxygen to glucose A-V differences known as the oxygen glucose index (OGI; equation (2)). Additionally, while previous PET-based reports of AG have only considered glucose metabolism,^17,18,22,23^ cross-brain blood sampling permits the easy inclusion of lactate, and thus the consideration of total carbohydrate metabolism, with the oxygen carbohydrate index (OCI; equation (3)).

#### Interpreting metrics of aerobic glycolysis

OGI quantifies the ratio of oxygen consumption relative to glucose (unitless) such that a decrease in this value (below 6) indicates greater glucose consumption relative to oxygen, that is, AG. A value of 6 is the theoretical maximum for OGI and indicates that all glucose is oxidized given the 1:6 stoichiometric ratio of glucose to oxygen. If a value >6 occurs, this suggests the complete oxidation of glucose plus other fuel sources that were unaccounted for. Theoretically, if the brain was exclusively consuming glucose via AG then we would expect an OGI approaching 0. OCI is interpreted similarly, that is, an absence of AG when OCI is 6. The inclusion of lactate with OCI can help explain an OGI <6 via glycolytic lactate production and release from the brain, whereby an OCI that is higher than OGI suggests such a scenario, due to a reduced denominator. Additionally, when circulating lactate is elevated it’s oxidative metabolism can account for 30%–50% of total brain metabolism,^43–45^ emphasizing its relevancy to include when assessing oxidative carbohydrate metabolism.

Finally, the percent of glucose/carbohydrate consumed, that is, not accounted for by oxygen consumption, and may be presumed to be undergoing aerobic glycolysis, was determined via equations (4) and (5).

### Quantification and statistical analysis

Outliers were removed if they were 1.5-times outside the interquartile range for OGI and OCI resulting in removal of 16 and 14 subjects, respectively. In 5 of the 17 studies (*n* = 36, 15%), multiple baseline samples were taken throughout the protocol, in which case values were averaged (coefficient of variation of 12%) within a participant to obtain a more robust measure of resting arteriovenous differences, OGI and OCI. Summary data comprising average values for arteriovenous differences, extraction fractions, and metrics of AG were calculated in age categories 19–24, 25–29, 30–34, 35–45, and 60+ years and displayed in violin plots for %AG and %AG_carb_ and reported in Table S2 for all measures.

We then correlated the extraction fractions, arteriovenous differences, and metrics of AG (OGI, OCI) with subject age to generate a linear regression of each relationship. Unless otherwise stated, all relationships were determined by linear mixed effects model with age as a fixed effect and study as a random effect using the “*lme4*” package in R (R Statistical Software v4.4.1; R Core Team 2024). The equation of the line, *p* value, and number of individual data-points are indicated in the figure. If a significant relationship (*p* < 0.05) were to occur, the slope was used to establish the rate of change in AG with aging such that a positive (*increasing*) slope indicates *decreasing* AG. We determined the strength of evidence in favor of either the null hypothesis or that AG changes with age from the Bayes factor (BF_10_) for the regression of OGI and age using the “*brms*” package in R. Priors for the intercept and slope were informed by PET-based estimates in 19–45-year-old participants reported by Goyal et al.^17^

A separate linear mixed model was run on males and females to investigate the effect of sex on aging and cerebral glucose metabolism for OGI and OCI, whereby both age and sex were considered fixed effects, and study as a random effect. Summary data split into age categories present values expressed as the mean ± standard deviation and differences between age groups were assessed with an ANOVA. Additionally, equivalence testing was done on the age-group data for %AG and %AG_carb_ using two one sided tests (“*TOST*” package in R) with the null hypotheses that the change in %AG or %AG_carb_ is either <−5% or >5% of the 19–24 age range. If both hypotheses are rejected at *p* < 0.05 then we concluded AG did not change with age compared to 19–24 years old.

To account for the potential influence of brain activation, we conducted a supplemental weighted regression analysis. Here, we applied an exponential decay weighting scheme to variables known to be sensitive to mental stimulation and thus reflective of an activated state. The variables included in this analysis were: (1) oxygen extraction fraction (OEF%), as it decreases locally^46^ and globally^19^ during brain activation and stress in response to elevated cerebral blood flow (CBF); (2) arterial PCO_2_ (PaCO_2_), as PaCO_2_ declines during mental stress and is the strongest determinant of global CBF^47,48^ and has a direct influence on OGI^49^; (3) arterial lactate, as it increases during mental and physical stress and provides the brain with oxidizable carbon substrate and thus contributes to oxygen consumption^43,44^; and (4) venous lactate, as lactate efflux from the brain increases during mental stimulation.^50,51^ For each of these variables, weights were assigned based on the distance from the median, with greater down-weighting of values that deviated substantially from the interquartile range (exponential decay). The individual weights were averaged into a composite weight, which was then incorporated into a linear mixed-effects model predicting OGI/OCI with age, including study as a random effect. The findings from this model should be considered exploratory and interpreted cautiously as some of the covariates used to account for brain activation (e.g. OEF, lactate ratios) are mathematically related to OGI/OCI. This may introduce endogeneity, where predictors and outcomes are not fully independent.

(1) $$Extraction\;\;Fraction\left(\%\right)=\frac{\left(A_{X}-V_{X}\right)}{A_{X}}\times 100\%$$

(2) $$OGI=\frac{\left(A-V_{O2}\right)}{\left(A-V_{glc}\right)}$$

(3) $$OCI=\frac{\left(A-V_{O2}\right)}{\left(A-V_{glc})+(\frac{1}{2}A-V_{lac}\right)}$$

(4) $$\%AG=\left(1-\frac{OGI}{6}\right)\times 100\%$$

(5) $$\%AG_{carb}=\left(1-\frac{OCI}{6}\right)\times 100\%$$

*A_X_* and *V_X_* represent the arterial and venous concentrations for oxygen (O_2_), glucose (glc), and lactate (lac), respectively.

## Results

### Study demographics

Across 17 studies, direct measures of cross-brain cerebral substrate and oxygen concentrations were obtained in 239 adults between ages 19 and 45 (*n* = 239, mean 26.4 ± 5.1 years), with an additional nine older subjects >53 years old (66.4 ± 6.7 years) for exploratory analysis in elderly (see Figure S1 for age histogram). After removing statistical outliers, we had 224/226 (OGI/OCI) subjects between 19 and 45 and eight subjects >60 years. Due to the scarcity of cross-brain data in individuals over 60 years of age in this dataset, and thus the risk for biasing model parameters, we have only included data from 19 to 45 years old in the results presented here. We include results for the 60+ age category in the presentation and statistical analysis of age group means in violin plots and the summary table (Table S2). Linear models including the total data set (age 19–77 years) are also presented in the Supplemental Material. Outliers were removed as outlined in methods and the resultant *n* for each variable and age category displayed in the figure. Resting baseline samples from each study were used to investigate basal oxygen, glucose, and lactate metabolism in normal aging, first assessing the change in individual substrate extraction fraction, then deriving metrics of AG. Lactate is included here for a more complete account of cerebral oxidative carbohydrate metabolism.

### Whole brain oxygen and carbohydrate extraction fraction are stable throughout adulthood

**Figure 1.**
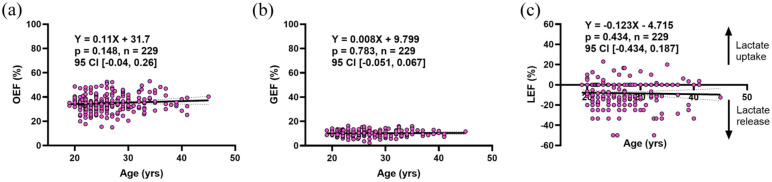
Stability of oxygen, glucose, and lactate extraction/release across the resting brain during adulthood. OEF (a), GEF (b), and LEF (c) do not change with age from 19 to 45 years old. GEF (10.3% ± 2.5%) remains lower than OEF (35.0% ± 6.9%), while LEF indicates a net release of lactate (−8.3% ± 12.3%). Equations of the line and statistics are displayed in each respective panel. All results were determined by a linear mixed effects model with age as a fixed effect and study as a random effect. Dotted lines represent the 95% confidence interval. OEF: oxygen extraction fraction; GEF: glucose extraction fraction; LEF: lactate extraction fraction.

Before examining relative rates of metabolism for oxygen and carbohydrates, we determined if any changes occur in independent substrate extraction with age. Previous studies of cerebral blood flow (CBF) and cerebral metabolic rates of oxygen (CMRO_2_) have demonstrated that a reduction in these variables can result in a compensatory increase of oxygen extraction fraction.^8,52,53^ This may have relevancy in aging given the observed reductions in CBF.^7,8^ Thus, the cross-brain extraction fraction for oxygen, glucose, and lactate (OEF%, GEF%, and LEF%, respectively) was calculated and plotted by age in Figure 1. No change in the extraction fraction of substrates was observed in the 19–45 age range. OEF% (35.0% ± 6.9%) remains significantly higher than GEF% (10.2% ± 2.5%, *p* < 0.001). LEF% is negative (−8.3% ± 12.3%) which agrees with previous reports of a net lactate release in the resting brain.^44,54^

### Substrate arteriovenous differences and the relative change with oxygen are stable during adulthood

**Figure 2.**
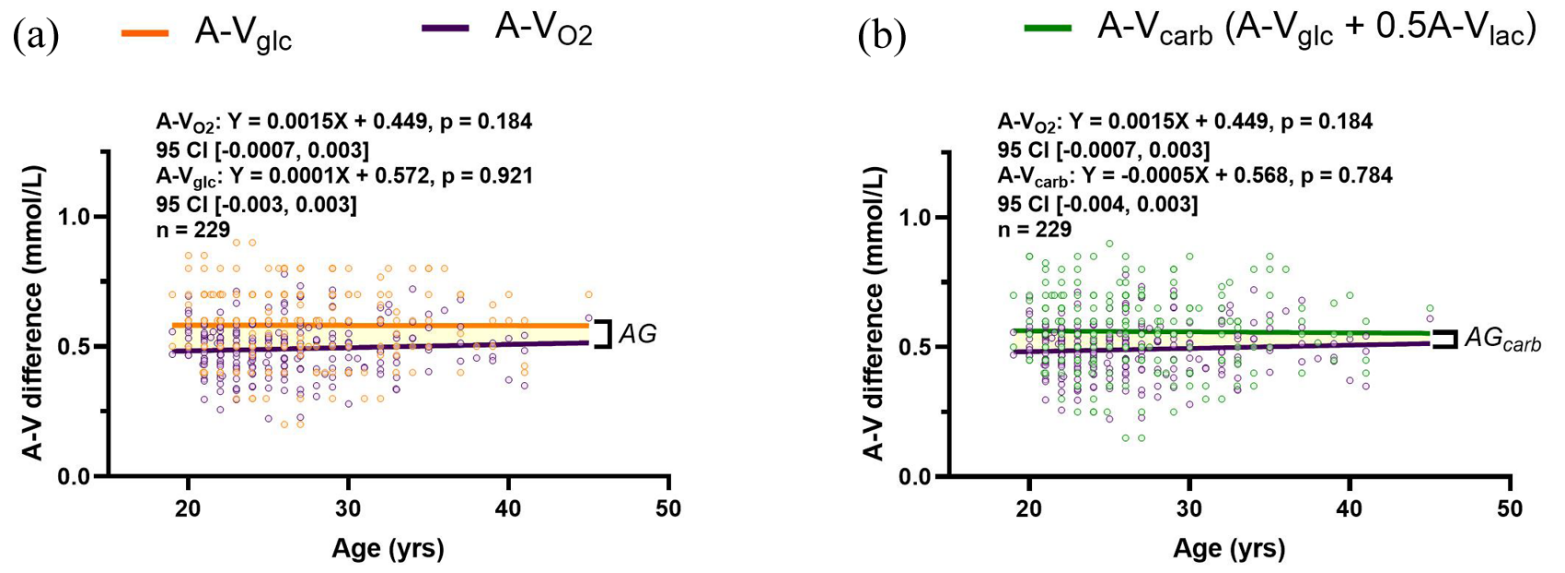
Substrate arteriovenous differences and the relative change with oxygen are stable during adulthood. The linear regression for the A-V difference of oxygen (purple line) and glucose (orange line) were plotted to depict the difference relative to each other, indicated by the highlighted region (a). The same plot was generated with total carbohydrate A-V differences ((A-V)_carb_ = (A-V)_glc_ + ½(A-V)_lac_, green line) and A-V_O2_ (b). In both figures the highlighted region is a visual representation of aerobic glycolysis (AG or AG_carb_). No change in cross-brain differences occurs with age. The slopes of the lines do not differ between A-V_O2_ and A-V_glc_ (*p* = 0.496) and A-V_O2_ and A-V_carb_ (*p* = 0.434), as determined by a linear mixed model assessing the interaction of age on A-V differences of glucose and carbohydrates to oxygen, respectively. Equations of the line and statistics are displayed in each respective panel, and were determined by a linear mixed effects model with age as a fixed effect and study as a random effect. A-V: arteriovenous.

The relationship between carbohydrate (glucose + lactate) and oxygen extraction across the brain was assessed to determine the magnitude of AG across adulthood. The difference in arterial and internal jugular vein concentrations were determined for oxygen (A-V_O2_), glucose (A-V_glc_), and lactate (A-V_lac_). Oxygen and lactate are scaled to their respective stoichiometric ratio for glucose such that A-V_O2_ was divided by 6 and A-V_lac_ by 2. This was done to facilitate the stoichiometric visualization of how glucose and total carbohydrate metabolism change relative to oxygen. The existence of AG is demonstrated by a greater cross-brain difference of glucose relative to oxygen (0.58 ± 0.14 vs 0.49 ± 0.10 mmol/L, *p* < 0.001) and represented by the highlighted region in Figure 2(a); that is, a closer gap between O_2_ and glucose extraction represents less AG. The total carbohydrate arteriovenous difference (A-V_carb_), while slightly lower than A-V_glc_ (0.56 ± 0.15 vs 0.58 ± 0.14 mmol/L, *p* = 0.116), remains higher than A-V_O2_ (*p* < 0.001), as depicted by the highlighted region in Figure 2(b). A linear regression for arteriovenous differences of oxygen, glucose, lactate, and total carbohydrates reveal no change with age and similarly no difference exists between the slope of each line. A slight narrowing of the gap between A-V_glc_/A-V_carb_ and A-V_O2_ may suggest the possibility of reduced AG (when considering total carbohydrate uptake) by late adulthood, although this is not statistically significant.

### Cross-brain oxygen-to-carbohydrate ratios are stable during adulthood and between sexes

**Figure 3.**
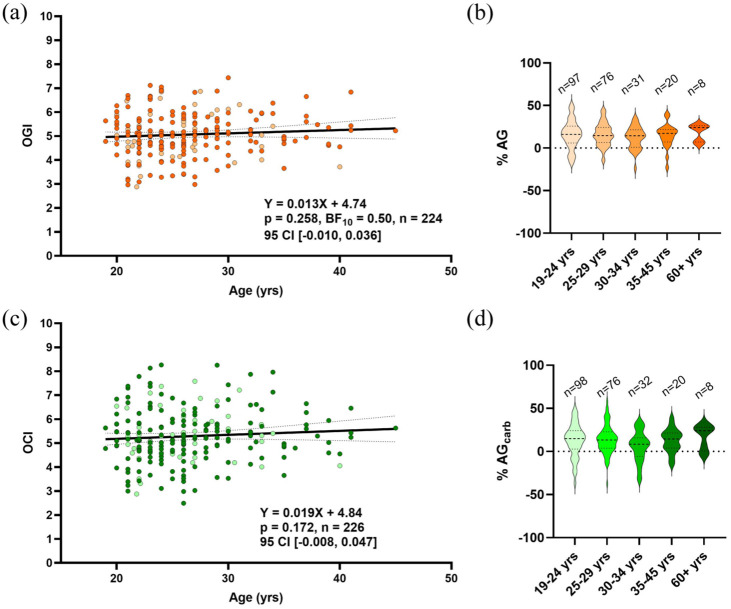
Stability in cross-brain oxygen to glucose or carbohydrate ratios with age and sex. Aerobic glycolysis is quantified by OGI = (A-V)_O2_/(A-V)_glc_ and OCI = (A-V)_O2_/((A-V)_glc_ + ½(A-V)_lac_). No effect of age was observed for both OGI (a) and OCI (c) for healthy subjects ranging from 19 to 45 years old. Results were determined by linear mixed effects model with age as a fixed effect and study as a random effect. Dotted lines represent the 95% confidence interval (95% CI). Sex is indicated by color shading, with no observed difference in OGI with age between males and females (light shading, *n* = 53) determined by a linear model including sex as a covariate (main effect of sex *p* = 0.749, interaction *p* = 0.712), and likewise for OCI (main effect of sex = 0.934, interaction = 0.951). (b) Depicts what percent of glucose metabolism is in excess of oxygen (%AG) in each age category, including 60+ years, based off OGI results. (d) Depicts what percent of carbohydrate metabolism is in excess of oxygen (%AG_carb_). No difference across age categories was detected, determined by ANOVA (*p* = 0.271 and 0.217, respectively). In the violin plots, center dotted lines indicate the median and the lower and upper dotted lines indicate quartile 1 and quartile 3, respectively. OGI: oxygen to glucose ratio; OCI: oxygen to carbohydrate ratio.

We further examined the relationship between brain oxygen and substrate extraction during adulthood with oxygen-to-carbohydrate indices. AG can be directly quantified by the oxygen to glucose ratio (OGI) and the oxygen to carbohydrate ratio (OCI). These metrics take the A-V_O2_ difference and divide it by A-V_glc_ and total carbohydrate difference, respectively. An OGI of 6 indicates no AG given the 6:1 molar ratio of oxygen and glucose. Conversely, a value lower than 6 indicates the consumption of glucose in excess of oxygen, that is, AG. Due to net lactate release from the brain (Figure 1(c)), the average OGI was less than OCI from 19 to 45 years of age (5.06 ± 0.90 vs 5.29 ± 1.10, *p* = 0.019), while both below 6 verifying the existence of AG in the resting human brain. Linear regression did not reveal an age-related change in OGI (Figure 3(a)) or OCI (Figure 3(c)). For OGI, the Bayes factor (BF_10_ = 0.50) indicated that our data were twice as likely under the null hypothesis of no age effect between 19 and 45 years.

Male and female data points are indicated by color shading (females as light color), with no observed sex effect for both OGI and OCI with age. We have also expressed each OGI and OCI value in terms of %AG or %AG_carb_ (Figure 3(b) and (d), respectively), representing the proportion of glucose or total carbohydrate extraction that is in excess of oxygen extraction. This provides an intuitive representation of AG which is indicated by the observed OGI and OCI values. In these data, eight individuals over 60 years of age are included to suggest that OGI and OCI are stable throughout the healthy lifespan based on no difference in %AG/AG_carb_ between age groups (*p* = 0.687 and 0.217, respectively). Additionally, equivalence testing indicated that all age groups were statistically equivalent to the 19–24-year-old group within a ±5% margin for both metrics, except for %AG_carb_ in the 30–34 and 60+ age groups. For these two comparisons, equivalence could not be concluded; however, traditional null hypothesis tests were also non-significant (*p* = 0.062 and 0.428, respectively), suggesting no evidence of a meaningful difference.

Finally, the weighted linear mixed-effects model accounting for indicators of brain activation (OEF%, PaCO_2_, arterial, and venous lactate) raised the average OGI and OCI to 5.12 ± 1.0 and 5.35 ± 1.2, respectively. OGI did not significantly increase with age (slope = 0.009, *p* = 0.632, 95% CI [−0.030 to 0.048], *R*^2^ = 0.03) nor did OCI (slope = 0.003, *p* = 0.905, 95% CI [−0.054 to 0.048], *R*^2^ = 0.04). This supplementary analysis was performed to assess the robustness of our findings when reducing the influence of potentially activated physiological states. These results suggest that accounting for this mildly activated mental state does not alter the relationship of OGI and OCI with age, however, should be interpreted cautiously due to the interdependence of OGI/OCI on the chosen predictor variables.

Summary data for arteriovenous differences, extraction fractions, and metrics of AG can be found in the Supplemental Material (Table S2). No difference by age group exists for any measure.

## Discussion

Using direct measures of oxygen, glucose, and lactate extraction, we report the presence of AG in the resting human brain, which does not decrease in 239 healthy individuals ranging from 19 to 45 years of age, with preliminary evidence to suggest that this persists into late adulthood (age 60+). This indicates that a decline in resting global brain AG is likely not a feature of normal aging during early-mid adulthood.

These data are inconsistent with the metanalysis of PET-derived global cerebral metabolism that reported a distinct drop in glucose metabolism explained primarily by a loss of AG, with the largest drop between ages 20 and 40 years.^17^ While technical error also exists in invasive cross-brain blood sampling data, the mathematical assumptions, smaller sample size, and the variable source of cerebral metabolic rates of glucose and oxygen (CMRglc and CMRO_2_) obtained from the PET-derived results may explain these different outcomes. Herein, we discuss the potential physiological and methodological explanations for our divergent findings.

Brain glucose metabolism is most commonly quantified via fluorodeoxyglucose positron emission tomography (FDG–PET) which relies on the detection of gamma rays emitted from the decay of a radioisotope of glucose ([^18^F]FDG) to gain insight into both regional and whole brain glucose metabolism. Likewise, ^15^O-PET can be used to calculate regional and global measures of oxygen consumption via the infusion of [^15^O]O_2_, a radioisotope of oxygen. However, one major limitation of PET-derived measures of AG is the inability to simultaneously measure CMRglc and CMRO_2_; yet a reasonable estimate under resting conditions can be made by conducting ^15^O-PET in rapid succession with FDG–PET. Despite this, the metanalysis data reported by Goyal et al.^17^ holds additional error as each reported AG was calculated using global CMRglc and CMRO_2_ values from different participants enrolled in different studies. For example, of the 106 data points between age 19 and 80 years, 33 data points had measures of global CMRglc while the remainder had measures of only CMRO_2_. Moreover, in the 19–45 age range—where AG decreased most—only 18 participants had measures of CMRglc. This is in contrast to the current study which includes >220 participants in this age range with *simultaneous* quantitative whole brain measures of glucose and oxygen extraction. It is also of note that the CMRglc data reported by Goyal et al. was acquired from only two studies, both of which report a decrease in glucose metabolism with age.^55,56^ It is unclear why these particular studies were chosen given that many other studies observe no change in CMRglc with aging (reviewed in Cunnane et al.^10^).

PET-based measures require mathematical models and assumptions to obtain quantitative results, contributing to error particularly when comparing between individuals.^57–59^ For example, to account for the differing kinetics of glucose and FDG transport and phosphorylation, a correction factor known as the lumped constant (LC) is required. Differing values have historically been used in FDG–PET literature, indeed the LC has been updated since the genesis of FDG–PET from 0.42 in studies from ~1980^60,61^ to higher values around 0.8 today.^62–64^ Moreover, physiological variability such as blood glucose levels at the time of the scan and variation in insulin sensitivity will impact the uptake of FDG, as well as individual differences in hexokinase activity effecting entrapment in the neuron.^65^ If an incorrect LC is used this will dramatically alter the reported CMRglc, thus warranting concern for using a single population-wide value. Benveniste et al. demonstrated using the data from the aforementioned metanalysis^17^ that increasing the LC to updated values for the children abolished the difference in AG between early childhood and healthy adults.^66^ Lastly, the fact that the LC has increased in the last 40 years suggests that updates in FDG–PET procedures and technologies pose limitations when combining and comparing data obtained across several decades. For example, mean CMRglc at rest measured by PET-imaging has also doubled (from ~4 to 8 mg/100 g/min) from the 1980s to 2004.^10^ As it stands, individual variability in resting CMRO_2_ and CMRglc from different people, and different studies, may lead to erroneous results when combining them to quantify global brain AG.

As discussed above, PET-derived results rely on generalized assumptions such as the LC, as well as other simplified mathematical frameworks such as normalization methods and scaling to population means,^18^ compartmental models,^60^ and standardized uptake approaches.^58^ Contrary to this, arteriovenous sampling directly measures the concentration differences of metabolites and gases across the brain, without reliance on predefined assumptions about tracer kinetics or metabolic uniformity. This methodology inherently permits (and perhaps emphasizes) interindividual variability in all physiological contributors to cerebral metabolism, including substrate delivery, enzymatic turnover, and tissue extraction. Indeed, there is notable variability present in our results and within the literature for metrics of cerebral metabolism quantified from cross-brain blood sampling. For example, the individual study 95% confidence intervals (95% CI) for OGI in a recent metanalysis range from 2.6 to 11.3, which includes data from 40 invasive cross-brain studies.^67^ The data included here has comparable 95% CI to the studies included in this metanalysis (Table S1 and Figure S5). Globally, this metanalysis reported a mean OGI of 5.46 (5.25–5.66) and here we found a mean of 5.19 (5.01–5.37) prior to outlier removal. Notably, despite our smaller sample size, the similarity in both study-level and global CIs suggests that the variability in our data is no greater, and potentially less, than that observed in the broader literature.^67^ While 85% of the variability in the previous metanalysis is attributed to study variation, we accounted for inter-study variability in our statistical models by including “study” as a random effect. Thus, we contend that what is presented here reflects true physiological variability—akin to the work by Blazey et al.—during adulthood and with that, no overall trend for change with aging.

The utility of PET-derived measures of cerebral metabolism are most evident when considering region specific differences, a feature that is lost with cross-brain arteriovenous blood sampling. This ability has led to the more accurate and timely diagnosis of neurodegenerative disease through detecting a reduction in glucose metabolism in certain brain regions, and furthermore the ability to track the progression of such diseases.^68,69^ In the same metanalysis by Goyal et al., a separate cohort was used to assess region-specific changes in AG. It was demonstrated that the cerebral topography of AG changes with age such that a reduction in AG is predominantly localized to regions of high AG during young adulthood and which possess increased gene expression related to synapse formation and growth.^22^ Despite the benefits of within participant regional comparisons, the limitations of deriving quantitative whole-brain values to assess inter-individual differences remain. Indeed it has also been demonstrated with PET that no such regional differences of AG exist in healthy humans at rest, which these authors attribute to the normalization process of previous work.^18,64^ However, reanalysis of the same dataset using a repeated measures approach revealed clear regional variation in AG, demonstrating how analytical choices, particularly whether or not subject-level variability is considered, can influence the interpretations of PET-based analysis.^70^

Despite the lack of mathematical assumptions when quantifying global cerebral metabolism from cross-brain blood sampling, limitations do exist beyond the inability to detect regional variation of cerebral metabolism. For example, the placement of the jugular catheter and the rate of sampling can influence jugular venous saturations due to contamination by extracerebral blood flow. However, sampling within 2 cm of the jugular bulb^42^ and at a rate <2 mL/min^71^ is suggested to yield blood containing <3% extracerebral contamination, otherwise an erroneous venous saturation may be measured. Moreover, it is possible that the observed lower mean OGI presented here reflects brain activation, stimulating AG and lowering OGI, in a participant who has just undergone invasive procedures. Any stress or anxiety associated with these procedures would result in a lower OGI than what is expected in a non-stimulated brain at rest.^19,46,72–74^ However, this factor can be overcome with time, and in the studies included here it was standard protocol to wait at least 1 h after line placement to begin resting measures.^74^ Regardless, the majority of the included studies had follow-up interventions such as intense exercise or drug infusions, possibly contributing to lasting anticipatory brain activation and low average OGI. Although the subjects included in this analysis may be mildly stimulated owing to the experimental procedures/interventions, the carbohydrate/oxygen ratios across the brain remain stable during aging. Additionally, accounting for blood-based variables that reflect brain activation, that is, OEF%, PaCO_2_ and lactate,^19,50,75^ increased average OGI and OCI but does not influence the relationship with age. Even with this additional analysis that accounts for biomarkers related to brain activation, OCI is ~5.4, indicating that ~10% of carbohydrate entering the brain is metabolized non-oxidatively within this age range. This stoichiometric evidence of AG in adulthood allows for the possibility for AG to approach zero with further aging, however, our data indicate there will be no meaningful decrease up to age 45, which is in contrast to the data of Goyal et al. whereby the vast majority of AG is lost by age 45, being completely absent by age 60.^17^

Both PET-imaging and cross-brain blood sampling possess strengths and weaknesses. However, we argue that direct cross-brain blood sampling is a stronger method for quantifying global AG due to the ability to simultaneously measure glucose and oxygen consumption without the need for generalized mathematical models. When measured this way, whole brain AG shows no meaningful change with healthy aging in adulthood (19–45 years). This is clinically relevant as brain glucose hypometabolism in adulthood is suggested to precede cognitive decline and other related neurodegenerative symptoms.^13–16^ Moreover, in eight healthy individuals over 60 years of age we show OGI and OCI are on average the same as the younger cohort (Figure S4 and Table S2). Due to the much smaller sample size in the elderly cohort of the work presented here, no strong conclusions can be made from these data; however, previous arteriovenous work demonstrated a notable, albeit non-significant, increase in oxygen-glucose metabolic ratios between a young (20.8 ± 0.4 years, *n* = 15) and old (71.0 ± 0.8 years, *n* = 26) cohort (means 5.5 ± 0.5 and 6.0 ± 0.2, respectfully).^9,25^ As it stands, more work must be done using consistent and direct measures of global cerebral metabolism in the healthy and cognitively impaired older adults to conclude if the drop in AG occurs after early adulthood or whether this is unique to pathological aging.

## Conclusion

Conclusions from PET-based data indicate that the loss of AG is a part of normal brain aging. Herein, using direct measures of glucose, lactate, and oxygen extraction across the brain in healthy humans aged 19–45 years old, we argue that global brain AG is likely maintained throughout healthy aging, particularly in early-mid adulthood. The discrepancy in the current findings may be explained by a difference in methodology and we contend that direct blood sampling provides a more robust and accurate measure of whole brain AG.

## Supplemental Material

sj-docx-3-jcb-10.1177_0271678X251399122 – Supplemental material for Brain aerobic glycolysis is stable during adulthood: Direct evidence from cross-brain blood sampling in 239 healthy adults Supplemental material, sj-docx-3-jcb-10.1177_0271678X251399122 for Brain aerobic glycolysis is stable during adulthood: Direct evidence from cross-brain blood sampling in 239 healthy adults by Jennifer S Duffy, Philip N Ainslie, Peter Rasmussen, David B MacLeod and Travis D Gibbons in Journal of Cerebral Blood Flow & Metabolism

sj-txt-1-jcb-10.1177_0271678X251399122 – Supplemental material for Brain aerobic glycolysis is stable during adulthood: Direct evidence from cross-brain blood sampling in 239 healthy adults Supplemental material, sj-txt-1-jcb-10.1177_0271678X251399122 for Brain aerobic glycolysis is stable during adulthood: Direct evidence from cross-brain blood sampling in 239 healthy adults by Jennifer S Duffy, Philip N Ainslie, Peter Rasmussen, David B MacLeod and Travis D Gibbons in Journal of Cerebral Blood Flow & Metabolism

sj-xlsx-2-jcb-10.1177_0271678X251399122 – Supplemental material for Brain aerobic glycolysis is stable during adulthood: Direct evidence from cross-brain blood sampling in 239 healthy adults Supplemental material, sj-xlsx-2-jcb-10.1177_0271678X251399122 for Brain aerobic glycolysis is stable during adulthood: Direct evidence from cross-brain blood sampling in 239 healthy adults by Jennifer S Duffy, Philip N Ainslie, Peter Rasmussen, David B MacLeod and Travis D Gibbons in Journal of Cerebral Blood Flow & Metabolism
