# Supplementary material for: Brain aerobic glycolysis is stable during adulthood: Direct evidence from cross-brain blood sampling in 239 healthy adults
Source: J Cereb Blood Flow Metab. 2026 Jan 8:0271678X251399122. Online ahead of print. doi: 10.1177/0271678X251399122 (PMC12783036; doi:10.1177/0271678X251399122)
Supplement: sj-docx-3-jcb-10.1177_0271678X251399122 – Supplemental material for Brain aerobic glycolysis is stable during adulthood: Direct evidence from cross-brain blood sampling in 239 healthy adults [file sj-docx-3-jcb-10.1177_0271678X251399122.docx]

**Supplemental Material:**

**
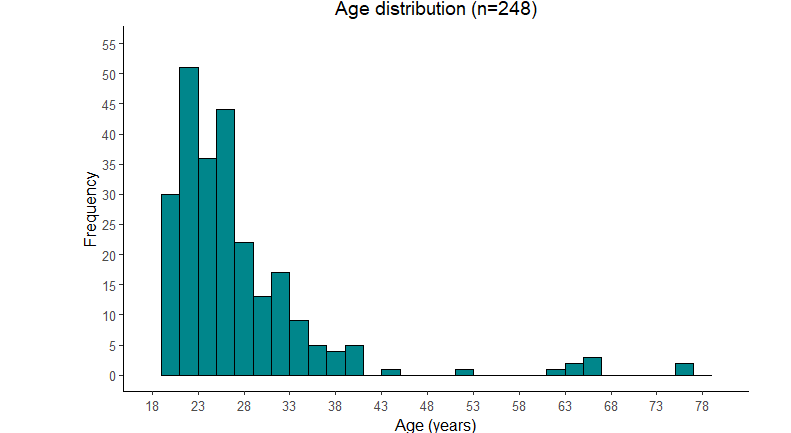
**

**Figure S1.** Age distribution for 248 individual subjects between age 19-77 whereby cross-brain (arterial and internal jugular vein) blood samples were obtained at rest. Due to the dearth of data between ages 45-60 yrs, linear models were established separately for the 19-45 and 19-77 age range, with the later presented in the Supplementary Material.

| **Study identifier** | **Date and Author** | **Purpose** | **Mean OGI [95 CI]** | **n** | **Outliers** | |
| --- | --- | --- | --- | --- | --- | --- |
|  |  |  |  |  | **OGI** | **OCI** |
| UBC1 | Bain et al. 2020 | To investigate the role of respiratory alkalosis on cerebral metabolic and inflammatory responses during passive heat stress | 4.8 [4.1, 5.5] | 4 (0F) | 0 | 0 |
| UBC2 | Smith et al. 2014 | To assess cerebral hemodynamics and metabolism at sea level and high altitude | 5.6 [4.8, 6.4] | 11 (1F) | 1 | 0 |
| UBC3 | Hoiland et al. 2020 | To assess the role of nitric oxide on neurovascular coupling via isovolumic hemodilution | 4.9 [4.5, 5.3] | 11 (0F) | 0 | 0 |
| UBC4 | Hoiland et al. 2022 | To assess the role of nitric oxide on cerebrovascular reactivity | 4.9 [4.4, 5.4] | 12 (0F) | 0 | 0 |
| UBC5 | Tymko et al. 2024 | To assess the role of cerebral sympathetic nervous activity in exercise-related cerebrovascular changes | 5.3 [4.6, 6.0] | 12 (5F) | 1 | 2 |
| UBC6 | Gibbons et al. 2024 | To examine cross-brain inflammatory, heamatological, neurotrophic responses to exercise at high altitude. | 5.1 [4.5, 5.7] | 12 (6F) | 0 | 0 |
| UBC7 | unpublished | To study the effect of combined hypercapnia, hypoxia, and hyperthermia (simulated avalanche burial) on cerebrovascular, metabolic, and inflammatory markers. | 5.8 [4.8, 6.9] | 14 (6F) | 2 | 3 |
| UBC8 | Koep et al. (in review) | To examine cerebral substrate utilization under various physiochemical states | 5.4 [5.0, 5.7] | 13 (6F) | 0 | 0 |
| MRL1 | Rasmussen et al. 2007 | To establish how well frontal lobe near-infrared spectroscopy follows capillary haemoglobin saturation | 5.7 [5.2, 6.3] | 11 (0F) | 0 | 0 |
| MRL2 | Rasmussen et al. 2010 | To investigate the fate of extra carbohydrate uptake by the brain during exercise | 6.0 [5.3, 6.6] | 15 (0F) | 4 | 4 |
| MRL3 | Overgaard et al. 2012 | To evaluate cerebral lactate uptake and release during exercise and hypoxia | 4.4 [4.1, 4.8] | 8 (2F) | 0 | 0 |
| MRL4 | Sørensen et al. 2012 | To establish the role of postoperative norepinephrine administration on near-infrared spectroscopy measured frontal lobe oxygenation | 5.2 [4.4, 5.9] | 13 (0F) | 0 | 0 |
| MRL5 | Fisher et al. 2013 | To assess differences of cerebral perfusion and metabolism during exercise between young and elderly individuals | 5.3 [4.5, 6.2] | 19 (9 old) | 1 | 1 |
| MRL6 | Rokamp et al. 2017 | To test whether the 46G > A (G16R) single nucleotide polymorphism of the β2-adrenergic receptor gene (ADRB2) influences the metabolic and cerebrovascular responses to administration of adrenaline | 4.7 [4.1, 5.2] | 37 | 5 | 2 |
| MRL7 | Siebenmann et al. 2021 | To establish the role of arterial lactate on cerebral lactate uptake during exercise | 5.4 [4.8, 6.0] | 8 (0F) | 0 | 0 |
| DUKE1 | unpublished | For calibration and validation of devices during FDA approval process | 5.1 [4.8, 5.3] | 29 (14F) | 0 | 0 |
| DUKE2 | unpublished | For calibration and validation of devices during FDA approval process | 5.0 [4.2, 5.8] | 19 (14F) | 2 | 2 |
| **Total** |  |  | 5.2 [5.0, 5.4] | 248 (55F) | 16 | 14 |

**Table S2.** Summary table of studies included in this analysis with n representing number of participants prior to outlier exclusion. UBC; University of British Columbia, MRL; Muscle Research Laboratory, DUKE; Duke University Medical Center. Mean OGI and 95 CI were calculated prior to outlier exclusion. *N/A as arterial and/or jugular venous concentrations for glucose and lactate were not provided to us, even if OGI/OCI was.

| **Age (yrs)** | **Extraction Fraction (%)** | | | **A-V Difference** | | | **OGI** | **%AG** | **OCI** | **%AG_carb_** |
| --- | --- | --- | --- | --- | --- | --- | --- | --- | --- | --- |
|  | **O_2_** | **Glc** | **Lac** | **O_2_ (mL/dL)** | **Glc (mmol/L)** | **Lac (mmol/L)** |  |  |  |  |
| **19-24** | 34.5 ± 6.2  n = 100 | 10.2 ± 2.3  n = 100 | -8.2 ± 12.0  n = 100 | 6.49 ± 1.20  n = 100 | 0.57 ± 0.13  n = 100 | -0.04 ± 0.07  n = 100 | 5.04 ± 0.99  n = 97 | 15.9 ± 16.7  n = 97 | 5.27 ± 1.18  n = 98 | 12.9 ± 19.1  n = 98 |
| **25-29** | 35.0 ± 7.8  n = 75 | 10.3 ± 2.6  n = 75 | -8.0 ± 11.6  n = 75 | 6.67 ± 1.56  n = 75 | 0.59 ± 0.14  n = 75 | -0.04 ± 0.07  n = 75 | 5.00 ± 0.85  n = 76 | 16.0 ± 13.6  n = 76 | 5.18 ± 1.05  n = 76 | 14.0 ± 16.5  n = 76 |
| **30-34** | 35.6 ± 7.0  n = 34 | 9.9 ± 2.8  n = 34 | -11.0 ± 14.2  n = 34 | 6.63 ± 1.37  n = 34 | 0.56 ± 0.14  n = 34 | -0.05 ± 0.07  n = 34 | 5.23 ± 0.82  n= 31 | 12.0 ± 13.1  n = 31 | 5.64 ± 1.14  n = 32 | 5.8 ± 17.8  n = 32 |
| **35-45** | 36.5 ± 6.4  n = 20 | 11.1 ± 2.5  n = 20 | -4.8 ± 13.0  n = 20 | 6.86 ± 1.20  n = 20 | 0.62 ± 0.13  n = 20 | -0.02 ± 0.07  n = 20 | 5.10 ± 0.78  n = 20 | 14.2 ± 14.4  n = 20 | 5.21 ± 0.78  n = 20 | 12.4 ± 13.9  n = 20 |
| **60+** | 38.3 ± 6.3  n = 7 | 10.8 ± 1.9  n = 7 | -2.0 ± 9.0  n = 7 | 7.39 ± 1.16  n = 7 | 0.67 ± 0.14  n = 7 | 0.00 ±0.08  n = 7 | 4.91 ± 0.58  n = 8 | 18.3 ± 9.8  n = 8 | 4.97 ± 0.85  n = 8 | 17.3 ± 14.0  n = 8 |

**Table S2.** Summary data comprising average values for arteriovenous differences, extraction fractions, and metrics of AG were calculated in age categories 19-24, 25-29, 30-34, 35-45, and 60+ years after outlier exclusion. Number of data points is indicated for each value. Data is expressed as means ± standard deviations. Note that average values for A-V_O2_ and A-V_lac_ are not scaled to their respective stoichiometric ratios of glucose, as is done in the linear regression with age.

**Figure S2. Nearly stable oxygen, glucose and lactate extraction/release across the resting brain during adulthood.** Oxygen extraction fraction (OEF; A) tends to increase slightly with age when including 7 individuals over the age of 60, glucose extraction fraction (GEF; B), and lactate extraction fraction (LEF; C) do not change with age from 19-77 years old. GEF (10.3 ± 2.5%) remains lower than OEF (35.1 ± 6.9%), while LEF indicates a net release of lactate (-8.1 ± 12.3%).

**Figure S3. Substrate arteriovenous differences are stable throughout adulthood and into elderly.** The linear regression for the arteriovenous (A-V) difference of oxygen (purple line) and glucose (orange line) were plotted to depict the difference relative to each other, indicated by the highlighted region (A). The same plot was generated with total carbohydrate A-V differences ((A-V)_carb_ = (A-V)_glc_ + ½(A-V)_lac_, green line) and A-V_O2_ (B). In both figures the highlighted region is a visual representation of aerobic glycolysis (AG or AG_carb_). No change in cross-brain differences occurs with age. The slopes of the lines do not differ between A-V_O2_ and A-V_glc_ (p=0.262) and A-V_O2_ and A-V_carb_ (p=0.258), as determined by a linear mixed model assessing the interaction of age on A-V differences of glucose and carbohydrates to oxygen respectively. All results were determined by a linear mixed effects model with age as a fixed effect and study as a random effect. Dotted lines represent the 95% confidence interval (95 CI). This figure includes 7 individuals over 60 years of age and replicates the findings for the 19-45 age range.

**Figure S4. Stability in cross-brain oxygen to glucose or carbohydrate ratios with age and sex.** Aerobic glycolysis can also be quantified by the oxygen to glucose (OGI = (A-V)_O2_/(A-V)_glc_) and carbohydrate (OCI = (A-V)_O2_/ [(A-V)_glc_ + ½(A-V)_lac_)] ratio. No effect of age was observed for both OGI (A) and OCI (B) for healthy subjects ranging from 19 to 77 years old. Results were determined by linear mixed effects model with age as a fixed effect and study as a random effect. Dotted lines represent the 95% confidence interval (95 CI). Sex is indicated by colour shading, with no observed difference in OGI with age between males (dark) and females (light, n = 53) as determined by a separate linear model considering sex as a covariate (main effect of sex p = 0.876, interaction p = 0.925), and likewise for OCI (main effect of sex = 0.605, interaction = 0.606). This figure includes 8 individuals over 60 years of age and replicates the findings for the 19-45 age range.


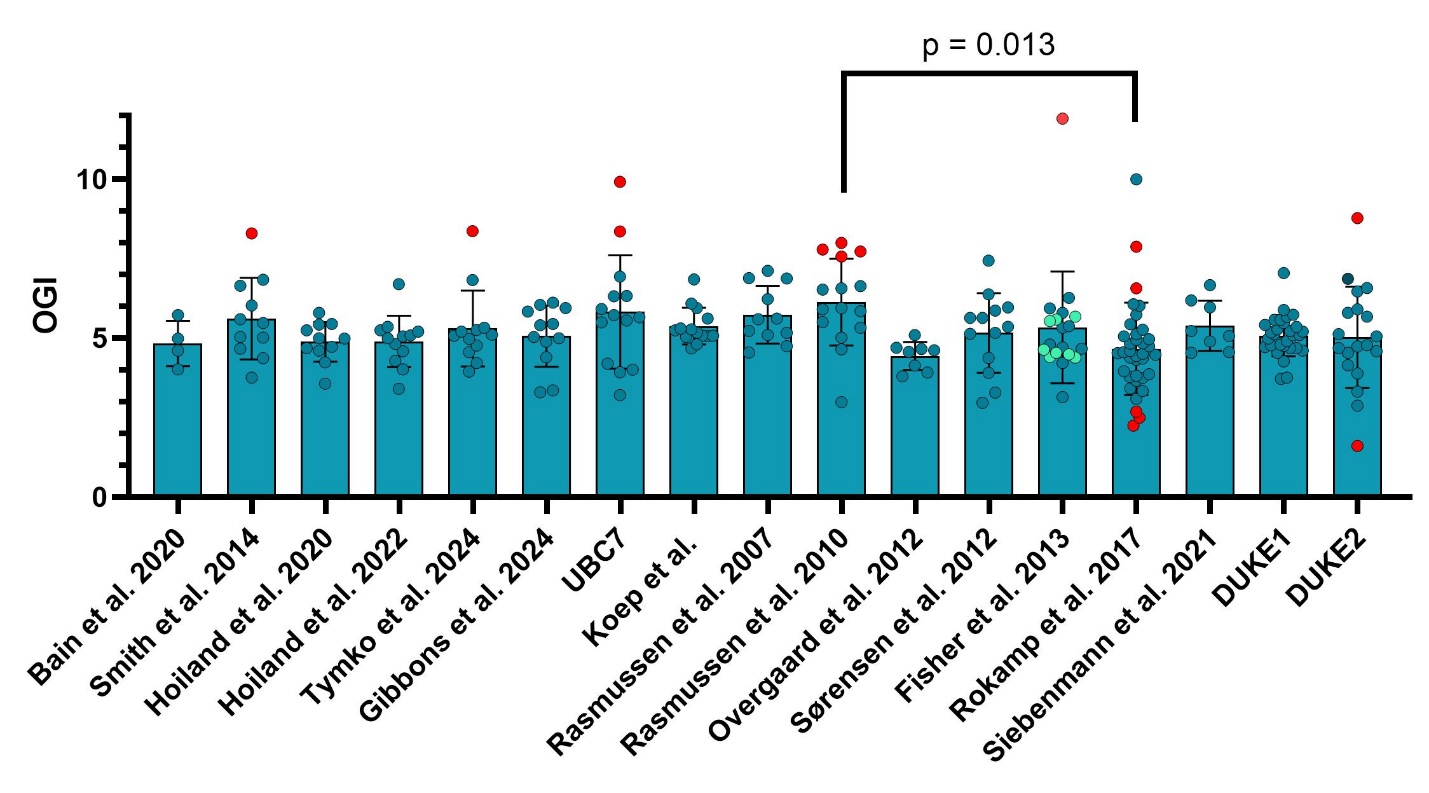


**Figure S5.** Average and individual OGI by study. Averages and ranges are homogenous with all but one pair lacking statistical differences. Red dots indicate statistical outliers while light green dots indicate individuals in the 60+ age category.
